# Incidence and risk factors for ventilator-associated pneumonia in Siriraj Hospital

**DOI:** 10.1186/cc11985

**Published:** 2013-03-19

**Authors:** O Chaiwat, S Nakaviroj

**Affiliations:** 1 Siriraj Hospital Mahidol University, Bangkok, Thailand

## Introduction

Ventilator-associated pneumonia (VAP) is a serious illness with substantial morbidity and mortality and increases the cost of hospital care. Even when bundles of care to prevent VAP have been implemented, the incidence of VAP was not dramatically improved. This study aims to determine the incidence and risk factors of VAP in the SICU of Siriraj Hospital.

## Methods

During a 1-year period, 228 patients admitted to the SICU were enrolled. All patients required ventilatory support longer than 48 hours. Data were collected by reviewing patient medical records including demographic data, onset of VAP, type of organisms, medication used, number of central venous catheters (CVC) used and blood transfusion. VAP outcomes were also reported.

## Results

VAP occurred in 21 patients (9.21%) or 8.21 per 1,000 ventilator-days. The onset of VAP was late in the majority of patients. The most common organism was *A. baumannii *(66%), followed by *P. aeuruginosa *(19%). Compared with non-VAP groups, patients in the VAP group had higher APACHE II score (18 vs. 13, *P *< 0.001), blood transfusion (95% vs. 75%, *P *= 0.04), numbers of CVC used (3 vs. 1, *P *< 0.001), muscle relaxant used (43% vs. 3%, *P *< 0.001) and steroid used (33% vs. 4%, *P *< 0.001). The VAP group also had a significantly higher number of intubation, reintubation and self-extubation. Multiple logistic regression showed that numbers of CVC, intubation and surgery, the use of muscle relaxant and steroid were independent risk factors for developing VAP. Ventilator days and ICU length of stay were longer in the VAP group (25 vs. 6 and 25 vs. 7 days, respectively). Lastly, the hospital mortality rate was significantly higher in the VAP group (33% vs. 12%, *P *= 0.008).

## Conclusion

The incidence of VAP was 9.2% in the SICU of Siriraj Hospital, which was comparable with previous reports. Bundles of care to prevent VAP should include weaning from a ventilator. Muscle relaxant and steroid should be administered according to strong indication. Meticulous care of the airway should be implemented as protocol in order to prevent complications that can result in the development of VAP.
